# ﻿The first record of the genus *Julolaelaps* Berlese (Acari, Mesostigmata, Laelapidae) in Republic of Korea and description of a new species on a captive giant African millipede (Spirostreptidae, *Archispirostreptus*)

**DOI:** 10.3897/zookeys.1180.109099

**Published:** 2023-09-15

**Authors:** Omid Joharchi, Seoyoung Keum, Chuleui Jung

**Affiliations:** 1 Agriculture Science and Technology Institute, Andong National University, Andong, Republic of Korea; 2 Institute of Environmental and Agricultural Biology (X-BIO), Tyumen State University, Tyumen, Russia; 3 Silkworm and Insect Management Center, Sangju, Republic of Korea; 4 Department of Plant medicals, Andong National University, Andong, Republic of Korea

**Keywords:** Arthropod-associated mites, Dermanyssoidea, East Palaearctic region, Gamasina, Indomalayan Realm, mesostigmatic mites, Parasitiformes, Spirostreptidae, taxonomy

## Abstract

This paper reports on a new species of mite of the genus *Julolaelaps* Berlese in Republic of Korea. Females and males of a new species, *Julolaelapsgigas***sp. nov.**, were collected on a captive giant African millipede, *Archispirostreptusgigas* (Spirostreptida: Spirostreptidae). The new species is described and illustrated based on morphological characters of the adult females and males and compared with known congeners. This new species is the first record of *Julolaelaps* from Republic of Korea. In addition, an updated key to all known species of the genus is presented.

## ﻿Introduction

Like most other arthropod groups, millipedes have attracted a mite fauna. The mites associated with millipedes are taxonomically and ecologically diverse, and about eighteen mite families have been recorded from the two orders Mesostigmata and Sarcoptiformes (Farfan and Klompen 2012).

Depending on the millipede and mite species, the nature of the symbiotic relationship can range from commensalism to obligate parasitism (Gerdeman et al. 2000; Farfan and Klompen 2012). Some of these mites may benefit their millipede hosts by helping to control populations of Acaridae, which sometimes become pests of captive millipedes (Swafford and Bond 2010; Farfan and Klompen 2012).

Ecological relationships such as these, with varying degrees of intimacy in the relationship between host animals and their associated mites, may represent a series of evolutionary stages from phoresy to parasitism. The genus *Julolaelaps* Berlese is one of the groups of Mesostigmata that includes 22 nominal species commonly associated with millipedes, mainly in Juliformia (Diplopoda) (Moraes et al. 2022), but some described species are associated with hosts in Polydesmida (Diplopoda) (Ishikawa 1986) and one species occasionally from the soil (Nemati et al. 2015). The genus is geographically distributed in Africa, the Middle East, East, South and Southeast Asia (Berlese 1916; Maes 1983; Fain 1987; Kontschán 2005; Uppstrom and Klompen 2005; Salmane and Telnov 2007; Moraza and Kazemi 2012; Nemati et al. 2015; Moraes et al. 2022). However, the nature of their symbiotic relationship (mutualism, commensalism and parasitism) with millipedes has not yet been confirmed (Salmane and Telnov 2007).

*Julolaelaps* was first established by Berlese (1916) for a small group of mites living on julids (Juliformes) belonging to the family Iphiopsididae. It was subsequently included in the Laelapidae-Hypoaspidinae by Vitzthum (1942: 763) and in the Laelapidae-Iphiopsidinae by Moraza and Kazemi (2012: 12). In this study, we follow Maes (1983) and Moraza and Kazemi (2012) in keeping the *Julolaelaps* as a separate genus of the family Laelapidae Canestrini, 1891, subfamily Iphiopsidinae Kramer, 1886.

Several authors have already recorded a collection of Laelapidae associated with insects and soil from the Republic of Korea (Kontschán et al. 2015, 2016; Keum et al. 2016, 2017; Joharchi et al. 2018, 2019; Oh et al. 2023). The present paper is part of a project that aims to increase the knowledge of the mite fauna of the Republic of Korea, particularly the poorly studied fauna of insect-associated species of mesostigmatic mites. Towards this aim, we herein describe a new species belonging to the genus *Julolaelaps* on the basis of female and male specimens collected on captive giant African millipede, *Archispirostreptusgigas* (Peters) (Spirostreptida: Spirostreptidae) in Republic of Korea. In addition, an update of the key of *Julolaelaps* species is presented to include the new species.

## ﻿Materials and methods

The millipedes were purchased for exhibition purposes and kept in a plastic cage with a sufficient amount of corroded oak sawdust. Mites were removed from the captive giant African millipede using a fine brush, cleared in lactic acid solution and mounted in PVA medium (Downs 1943). The line drawings and examinations of the specimens were performed with a Zeiss Axio Imager A2 compound microscope equipped with differential interference contrast optical systems and an attached Axiocam 506 color camera (Carl Zeiss, Germany). Most images were captured in stacks (with focal depth manually controlled). Selected images were combined using Helicon Focus 7.6.4 Pro (Helicon Soft Ltd, 2000). Digital drawings were prepared using Adobe Photoshop CS2 software based on the original pencil line drawings. Images and morphological measurements were taken via ZEN 2012 software (version 8.0). Measurements of structures are expressed as ranges (minimum–maximum) in micrometres (μm). The length and width of the dorsal shield were taken from the anterior to the posterior margins along the midline, and at level of *r4*, respectively. Length and width of the sternal shield were measured at the maximum length and broadest points, respectively. The length of the epigynal shield was measured along the midline from the anterior margin of the hyaline extension to the posterior margin of the shield, and its maximal widthwas measured posterior to genital setae *st5*. Leg length was measured from the base of the coxa to the apex of the tarsus (excluding the pre-tarsus). The nomenclature used for the dorsal idiosomal chaetotaxy follows that of Lindquist and Evans (1965), the notations for leg and palp setae follow those of Evans (1963a, b), and other anatomical structures mostly follow Evans and Till (1979). Notations for idiosomal pore-like structures (gland pores and poroids/lyrifissures) and peritrematal shield follow mostly Athias-Henriot (1971, 1975). The notations for pore-like structures on the sternal shield and for the peritrematal shield region also follow modifications and additions by Johnston and Moraza (1991). Holotype and paratypes of the new species were deposited at the National Institute of Biological Resources (NIBR), Republic of Korea.

## ﻿Results

### ﻿Genus Julolaelaps

Taxon classificationAnimaliaMesostigmataLaelapidae

Berlese

74184677-0EB4-50DB-B065-565362759728

Julolaelaps Berlese, 1916: 31. Type species Julolaelapsdispar Berlese, 1916, by original designation.
Hypoaspis (Julolaelaps) . – Ryke, 1959: 7.

#### Remarks.

The concept of *Julolaelaps* used here is based on that of Moraes et al. (2022). More information about the synonyms and nomenclatural history of the genus are available in Moraes et al. (2022: 316).

### ﻿ Julolaelaps gigas sp. nov.

Taxon classificationAnimaliaMesostigmataLaelapidae

EA0D6D37-B974-56BE-9137-8746BB42DFB4

https://zoobank.org/FDD68D96-90DF-4196-9DD5-377DCF03D633

Figs 1–5 , 6–9 , 10, 11 , 12–16

#### Material examined.

***Holotype*.** Republic of Korea • ♀; Sangju, Silkworm and Insect Management Center; 36°57'N, 128°15'E; 19 May 2020; S. Keum leg.; on captive giant African millipede, *Archispirostreptusgigas* (Spirostreptida: Spirostreptidae); NIBRIV 0000905722. ***Paratypes*.** Republic of Korea • 2♀ 2♂; same data as for holotype; NIBRIV 0000905723, NIBRIV 0000905724, NIBRIV 0000905725, NIBRIV 0000905726.

#### Diagnosis.

Dorsal shield smooth, without reticulation, bearing 22 pairs of minute acicular setae, setae *Z5* conspicuously longer than other setae on shield, podonotal and opisthonotal parts of dorsal shield slightly hypotrichous, setae in *z*- and *s/S-series* displaced laterally (on soft cuticle) and some missing (*z1*, z6, *J3*); posterior margin of shield somewhat truncated; dorsal shield reduced, not covering entire idiosoma, opisthogastric and lateral soft cuticle with 37 pairs of setae, including 23 pairs of *r*-*R*-*UR* setae. Sternal shield reduced, bearing only *st1* and *st2*, eroded posteriorly, *st3* and metasternal setae (*st4*) inserted on unsclerotized cuticle, epigynal shield drop-shaped, parapodal plates broadly thickened, extending behind coxae IV, endopodal plates III/IV broadly thickened, peritreme relatively long and rather broad, extending to anterior level of coxa II, cheliceral fixed digit with 2–3 teeth. Chaetotaxy of legs normal for free-living Laelapidae, except tibia III with nine setae (two posterolateral setae present) and genu IV with 10 setae (with two posterolateral setae). Male with separate sternogenital and anal shields, chelicerae with edentate digits including a regressed fixed digit and a stylet-like movable digit bordered by a very long spermatodactyl.

**Figures 1–5.**
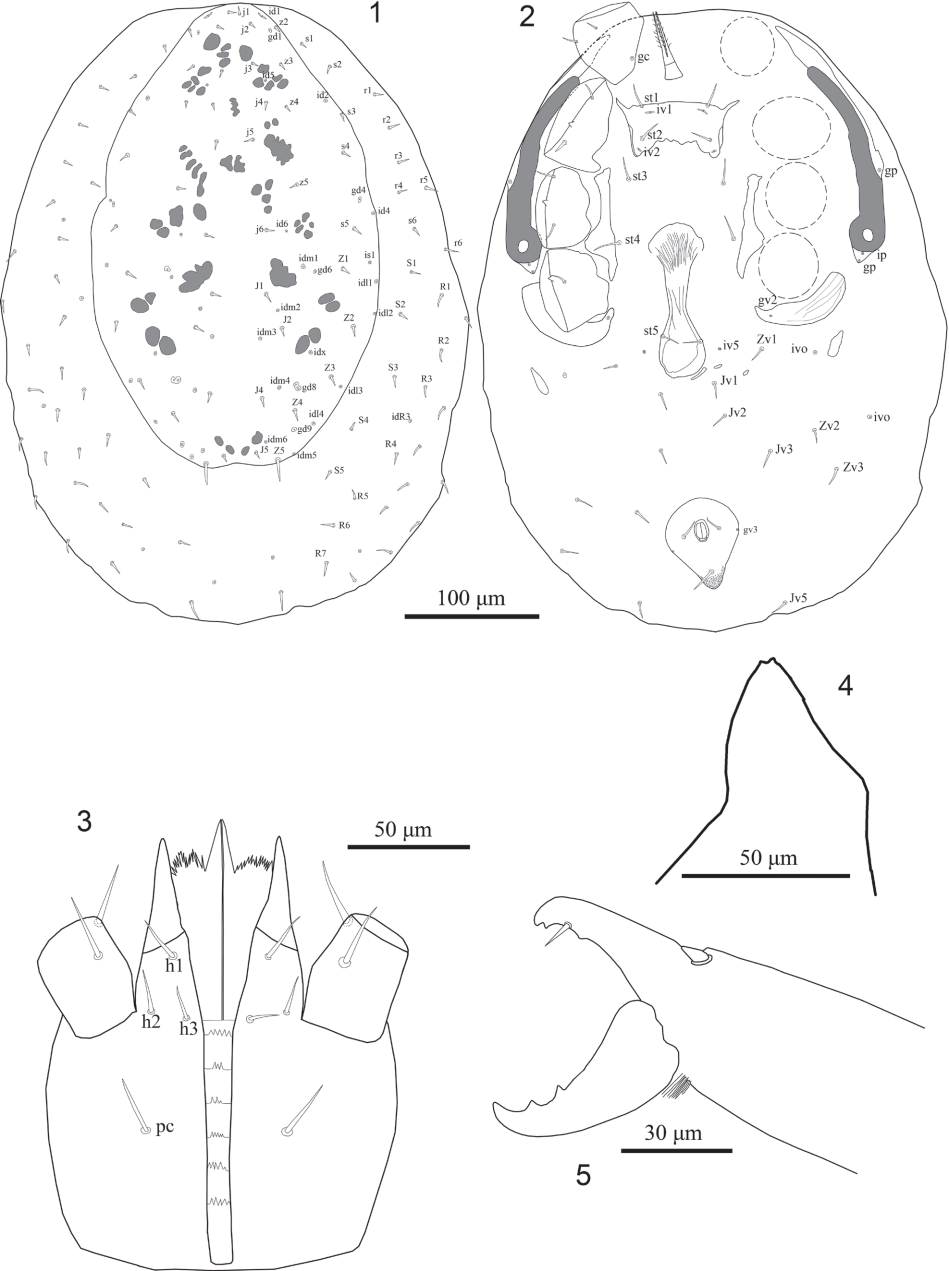
*Julolaelapsgigas* sp. nov., female, with symbols for chaetotactic notation of idiosomal setae **1** dorsal idiosoma **2** ventral idiosoma **3** subcapitulum **4** epistome **5** chelicera, lateral view.

#### Description.

**Figures 6–9.**
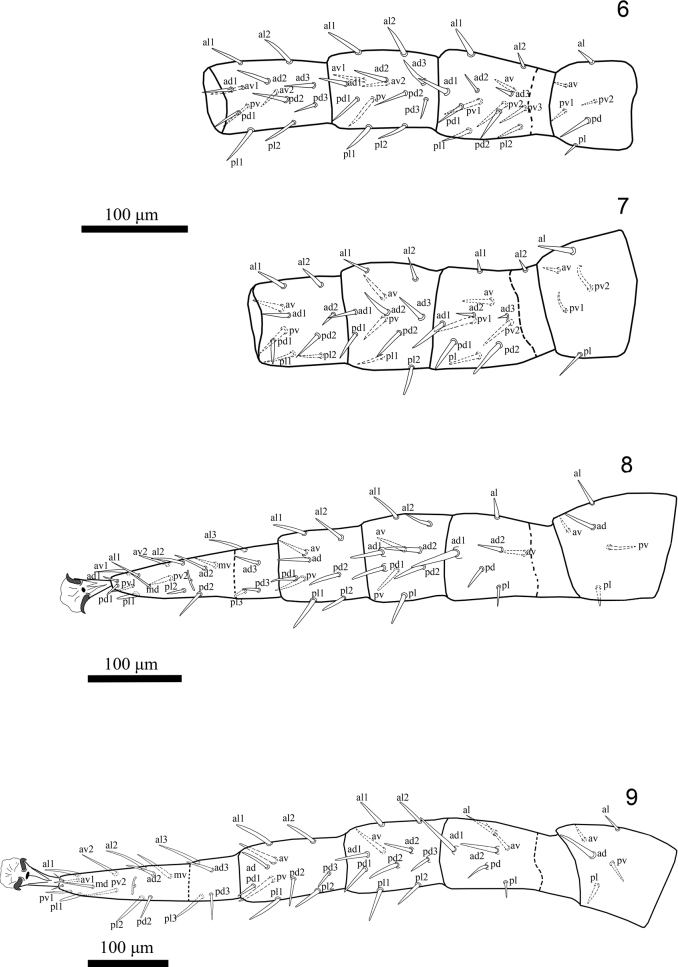
*Julolaelapsgigas* sp. nov., female, with symbols for chaetotactic notation of legs setae **6** leg I (trochanter-tibia, dorsal aspect) **7** leg II (trochanter-tibia, dorsal aspect) **8** leg III (trochanter-tarsus, dorsal aspect) **9** leg IV (trochanter-tibia, dorsal aspect).

**Female** (*N* = 3). Figs 1–9, 12–14. ***Dorsal idiosoma*** (Figs 1, 12). Length 906–925, width 620–649. Dorsal shield length 687–696, width 391–395, posteriorly somewhat truncate, reduced, not covering entire idiosoma, some dorsal setae left in the soft cuticle lateral to shield, becoming hypertrichous cuticle surrounding dorsal shield, without distinct reticulate ornamentation over whole surface; with 22 pairs of minute acicular setae, 13 pairs of podonotal setae, plus nine pairs of setae on lateral soft cuticle (*s1*, *s2*, *s6*, *r1-6*), nine pairs of opisthonotal setae, plus 21 pairs of setae on lateral soft cuticle (including *S1-5* and *R1-7*); *Zx*, unpaired or asymmetrical setae and *z1*, *z6*, *J3* absent, setae similar in length (9–15) and thicknesses; clonal setae *Z5* (26–31) longer than other setae on shield. Muscle insertions prominent as desclerotised circular patches. Dorsal shield with 22 pairs of discernible pore-like structures, including only five pairs of gland openings (*gd1*, *gd4*, *gd6*, *gd8*, *gd9*) and 17 pairs of poroids. Shape, position and relative length and thicknesses of setae shown in Figs 1, 12.

***Ventral idiosoma*** (Figs 2, 13). Tritosternum with paired pilose laciniae (105–113), short base 30–35 × 23–27 wide; presternal platelets absent. Sternal shield reduced (59–63 × 134–142 wide), shield with two pairs of smooth sternal setae [*st1*, *st2* (20–26)], and two pairs of poroids (*iv1*, *iv2*), eroded posteriorly, surface without reticulate ornamentation, smooth, *st3* (30–32) and metasternal setae (*st4*, 29–31) inserted on soft integument, metasternal poroids (*iv3*) apparently absent (Figs 2, 13), shield fused anterolaterally to narrow endopodal strip between coxae I and II, endopodal plates III/IV broadly thickened (Figs 2, 13). Epigynal shield drop-shaped, with an obvious (narrow) neck at level of coxae IV, protruding at level between setae *st5* and *Jv1*, width (65–70) and length (228–235), anterior and posterior margins rounded, with some irregular longitudinal and oblique lines, otherwise relatively smooth, bearing a pair of simple setae *st5* (20–23) inserted on lateral margins of shield, near level of posterior edge of coxae IV; paragenital poroids *iv5* located on soft cuticle lateral to shield near seta *st5* (Figs 2, 13). Anal shield subtriangular, rounded anteriorly, length 133–138, width 108–114, surface without reticulate ornamentation, smooth, para-anal setae (23–27) shorter than post-anal seta (30–35), cribrum well developed, with 3–4 irregular rows of spicules, each extending from cribrum to near base of post-anal setae; anal opening located at anterior level of shield; pair of glands *gv3* inserted on shield lateral margins, at level para-anal setae (Figs 2, 13). Soft opisthogastric cuticle surrounding epigynal and anal shields with one pair of suboval metapodal plates (38–43 long × 13–18 wide) and seven pairs of smooth setae (*Jv1–Jv3*, *Jv5*, *Zv1–Zv3*), setae more or less same in length (18–24) and thickness (Figs 2, 13). Peritreme relatively long and broad, extending to anterior level of coxa II, peritrematal shield narrow, slightly expanded anteriorly, each shield bearing three discernible pore-like structures, a gland pore *gp2* at level of coxa III, a lyrifissures *ip3* and a gland pore *gp3* on barely developed poststigmatic section (Figs 2, 13), anterior part of shield not fused with dorsal shield; parapodal platelets broadly thickened, extending behind coxae IV, bearing gland pore *gv2*; exopodal platelets absent (Figs 2, 13).

***Gnathosomal structures*** (Figs 3–5, 14). Epistome triangular, with pointed apex and smooth (Fig. 4). Hypostomal groove with six transverse rows of denticles, each row with 3–5 denticles, with smooth anterior and posterior transverse lines (Fig. 3). Hypostome with four pairs of smooth setae, *pc* (24–26) > h1 = h2 (18–21) > *h3* (14–16) (Fig. 3). Corniculi robust and horn-like, extending slightly beyond palpfemur. Internal malae with one pair of smooth median projections, flanked by lobes with fimbriate anterior margin; labrum with pilose surface; supralabral process not distinct. Chaetotaxy of palps: trochanter 2, femur 5, genu 6, tibia 14, tarsus 15, all setae smooth; palpfemur with seta *d3* thickened and *al* paddle-like; palpgenu with *al1* stout, blunt, *al2* thickened and spatulate; palptarsal apotele two-tined. Fixed digit of chelicera with 2–3 teeth and long pilus dentilis, dorsal cheliceral seta relatively thick and short, arthrodial membrane with a rounded flap and normal filaments; movable digit with two mid-sized teeth (Figs 5, 14).

***Insemination structures*.** Not seen, apparently unsclerotized.

***Legs*.** (Figs 6–9). Legs II and III short (600–650, 625–655), I and IV longer (657–680, 739–755). Chaetotaxy normal for free-living Laelapidae: Leg I (Fig. 6): coxa 0-0/1, 0/1-0, trochanter 1-0/1, 1/2-1, femur 2-3/1, 2/3-2, genu 2-3/2, 3/1-2, tibia 2-3/2, 3/1-2. Leg II (Fig. 7): coxa 0-0/1, 0/1-0, trochanter 1-0/1, 0/2-1, femur 2-3/1, 2/2-1, genu 2-3/1, 2/1-2, tibia 2-2/1, 2/1-2. Leg III (Fig. 8): coxa 0-0/1, 0/1-0, trochanter 1-1/1, 0/1-1, femur 1-2/1, 1/0-1 (*ad* thickened and inserted on small tubercles), genu 2-2/1, 2/1-1 (*ad1* and *pd1* inserted on small tubercles), tibia: 2-1/1, 2/1-2. Leg IV (Fig. 9): coxa 0-0/1, 0/0-0, trochanter 1-1/1, 0/1-1 (*ad* thickened), femur 1-2/1, 1/0-1 (*ad1* longest, *ad1* and *ad2* inserted on small tubercles), genu 2-2/1, 3/0-2, tibia 2-1/1, 3/1-2. Tarsi II-IV with 18 setae (3-3/2, 3/2-3 + *mv*, *md*); with some ventral and lateral setae thickened. All pretarsi with well-developed paired claws, rounded pulvilli and normal ambulacral stalk.

**Figures 10, 11.**
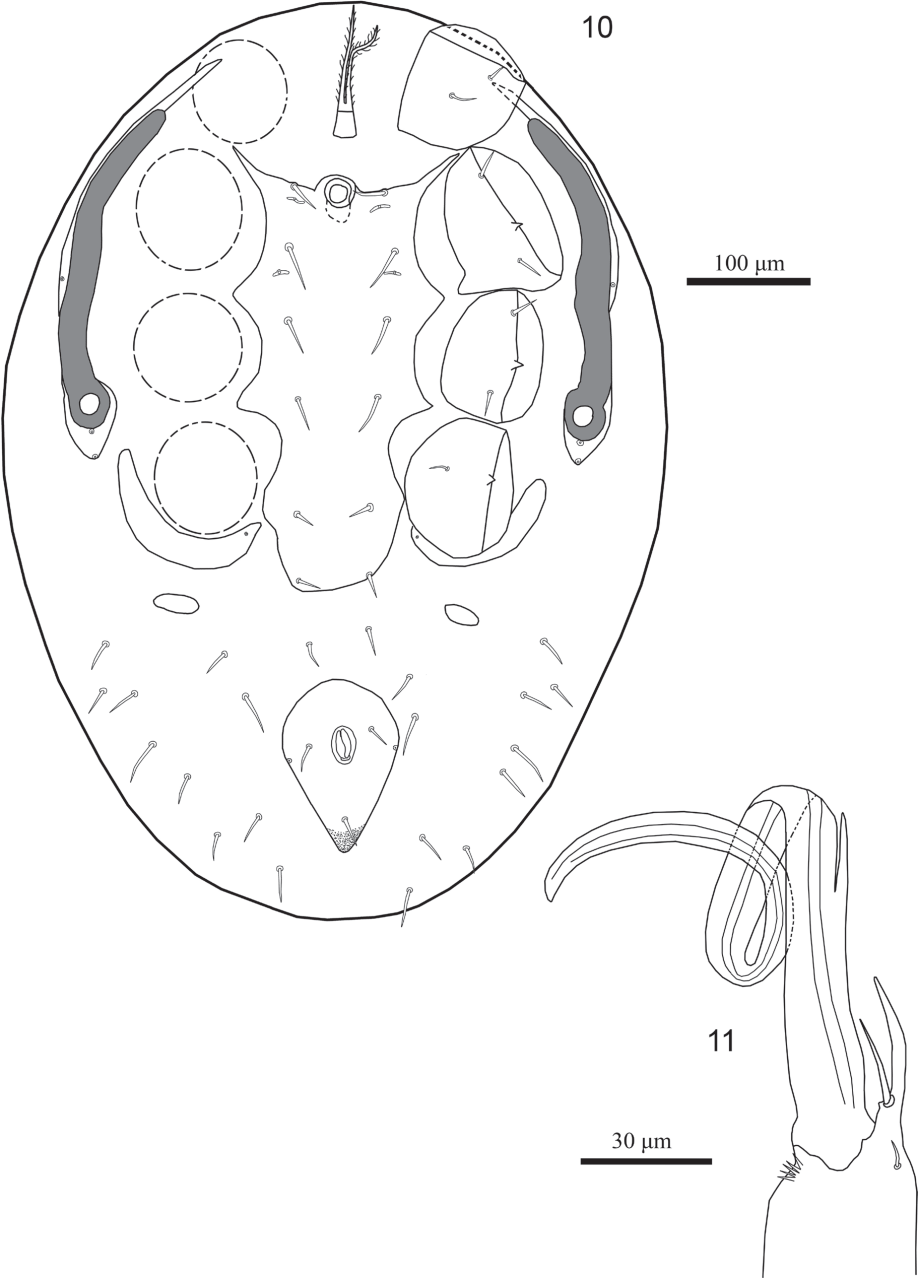
*Julolaelapsgigas* sp. nov., male **10** ventral idiosoma **11** chelicera, lateral view.

**Male** (*N* = 2). Figs 10, 11, 15, 16. ***Dorsal idiosoma*.** Length 760–768, width 525–530. Dorsal shield length 647–653, width 345–350; ornamentation and chaetotaxy as in female.

***Ventral idiosoma*.** (Figs 10, 15). Sternal, genital, endopodal, ventral shields fused into sternogenital shield, 327–333 long from anterior to posterior margins of shield, 175–180 wide at level of *st2*, 160–165 at *st3* level and 118–123 at *st5* level; shield with six pairs of simple sternal setae (*st1–5* and *Jv1*) (19–30), and two pairs of poroids; anal shield free, length 117–123, width 93–98, surface without reticulate ornamentation, smooth, post-anal seta (25–28) slightly longer than para-anals (18–23), cribrum well developed, with 3–4 irregular rows of spicules, each extending from cribrum to near base of post-anal setae; anal opening located at anterior level of shield; pair of glands *gv3* inserted on shield lateral margins, at level para-anal setae (Figs 10, 15). Soft opisthogastric and lateral cuticle with 10–12 pairs of setae. Peritremes, peritrematal shields and other ventral structures similar to those in female.

***Gnathosoma*.** (Figs 11, 16). Chelicerae with edentate digits including a regressed fixed digit and a stylet-like movable digit bordered by a long spermatodactyl, pilus dentilis long. Other gnathosomal structures similar to those in female.

***Legs*.** Chaetotaxy as in female.

#### Etymology.

The name of this species refers to its occurrence on giant African millipede of the species *Archispirostreptusgigas* (Spirostreptida: Spirostreptidae).

#### Differential diagnosis.

The female of the new species is unique within *Julolaelaps* because of its conspicuously reduced sternal shield, bearing only two pairs of sternal setae (*st1*, *st2*), eroded posteriorly and endopodal plates III/IV broadly thickened. In other features the new species is closest to *J.vandaelensis* Maes, 1983 in having a genital shield narrower than anal shield, peritreme almost reaching anterior margin of coxae II and dorsal shield with 22 pairs of setae. It is distinguished from this species by the uniform minute acicular dorsal setae and the reduced dorsal shield, not covering entire idiosoma, leaving an unsclerotised cuticle surrounding the dorsal shield.

**Figures 12–16.**
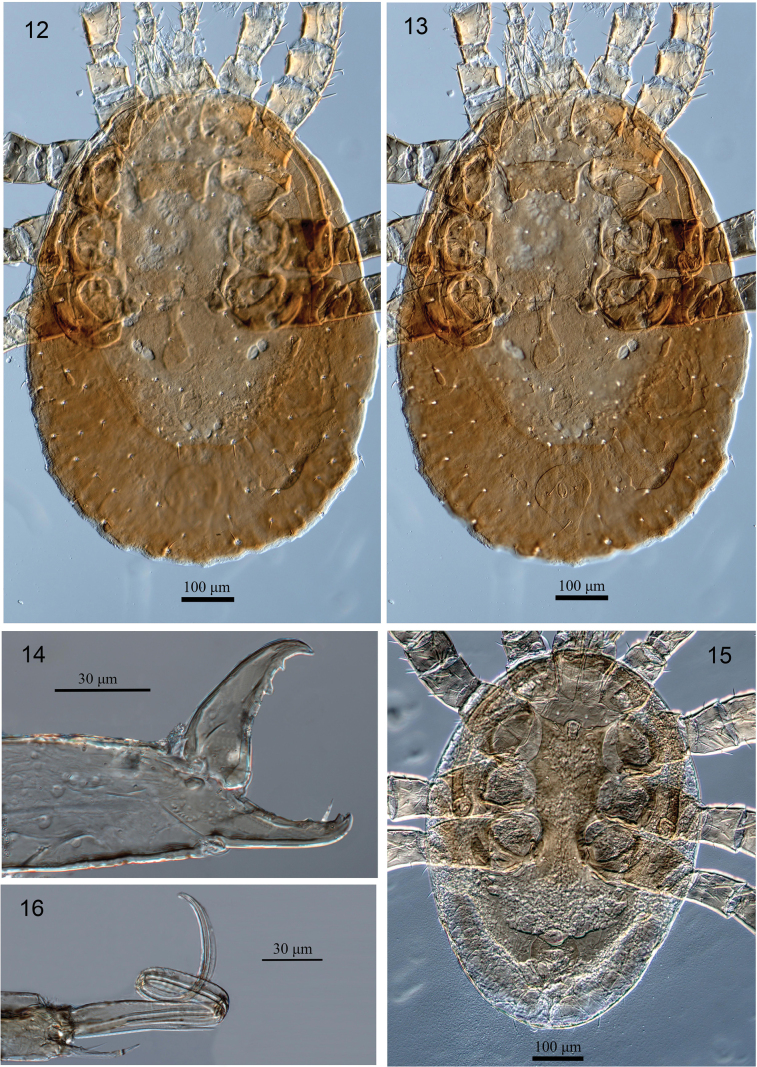
Differential Interference Contrast (DIC) micrographs of *Julolaelapsgigas* sp. nov., female **12** idiosoma in dorsal view **13** idiosoma in ventral view **14** chelicera in lateral view, male **15** idiosoma in ventral view **16** chelicera in lateral view.

## ﻿Discussion

Casanueva (1993) redefined the family Laelapidae based on phylogenetic evidence, merging the genera associated with millipedes (groups VI and VII, see Casanueva 1993) with the genera associated with Araneae and Crustacea (group VIII, fig. 7, p. 39), elevating the subfamily (Iphiopsidinae) to the level of the family (Iphiopsididae). According to Casanueva (1993), the regressive autopomorphy “absence of seta *av2* on tibia I, see Casanueva 1993 attribute #62” and “absence of seta *pl2* on genu IV, see Casanueva 1993 attribute #58” are the phylogenetic characters that define Iphiopsididae as a separate family from Laelapidae. Based on attributes #58 and #62, the placement of the new species (*Julolaelapsgigas*) in Iphiopsididae cannot be considered due to the presence of both setae *av2* and *pl2* on tibia I and genu IV, respectively.

Moraza and Kazemi (2012) recognized three species groups within the genus, but the inclusion of *Julolaelapsgigas* sp. nov. requires some changes to the diagnosis they introduced for each species group. Here we combine them into two species groups and call them simply as follows: *dispar* and *gigas* species groups. The *dispar* group includes species with largely complete dorsal setae complement, with more than 29 setae pairs (at least series *J* and *Z* complete), while the *gigas* species group is characterized by reduced dorsal setae, with at most 25 setae pairs (at least series *J* and/or *Z* incomplete). *Julolaelapsbuensis* Maes, 1983 was not included in the key because the dorsal of the idiosoma is not described due to poor fixation (Maes 1983). We have attempted to provide diagnostic characters for each species and to classify each species as either *dispar* or *gigas*. We stress that the information below was derived from the descriptions only, and most of these species are in need of redescription.

### ﻿Modified key to the species of *Julolaelaps* based on females (partly from Moraza and Kazemi 2012), with emendations to add *Julolaelapsgigas* sp. nov.

**Table:** 

1	Dorsal shield setation hypotrichous, with at most 25 pairs of setae, at least series *J* and/or *Z* incomplete (*gigas* species group)	**2**
–	Dorsal setation holotrichous or polytrichous, with more than 29 pairs of setae, series *J* and *Z* usually complete (*dispar* species group)	**15**
2	Sternal shield conspicuously reduced, bearing only two pairs of sternal setae (*st1*, *st2*), endopodal plates III/IV broadly thickened	***Julolaelapsgigas* sp. nov.**
–	Sternal shield well developed, bearing three pairs of sternal setae (*st1–3*), if endopodal plates III/IV present not broadly thickened	**3**
3	Epigynal shield drop-shaped, with an obvious (narrow) neck at level of coxae IV, narrower than anal shield; males with edentate chelicerae	**4**
–	Epigynal shield well developed, wider than anal shield, males having dentate chelicerae	**13**
4	Peritreme short, reaches at most to anterior level of coxa III	**5**
–	Peritreme long, reaches at least to level of coxa II	**10**
5	Dorsal shield conspicuously reduced, with nine pairs of setae (including three opisthonotal pairs)	***J.paucipilis* Fain, 1987**
–	Dorsal shield with more than nine pairs of setae	**6**
6	Dorsal shield with 14 pairs of setae (including four opisthonotal pairs)	**7**
–	Dorsal shield with 17–20 pairs of setae	**8**
7	Marginal setae on dorsal shield conspicuously longer than central setae; ventral setae simple; endopodal extension of sternal shield between coxae II and III well developed; epistome smooth	***J.madiakokoensis* Fain, 1987**
–	Marginal setae on dorsal shield as long as or slightly longer than central setae; endopodal extension of sternal shield between coxae II and III absent; epistome serrate	***J.idjwiensis* Fain, 1987**
8	Dorsal shield with 17 pairs of setae (including six opisthonotal pairs)	***J.peritremalis* Ryke, 1959**
–	Dorsal shield with 19–20 pairs of setae	**9**
9	Dorsal shield with 19 pairs of smooth setae (including four opisthonotal pairs), the central ones shorter than lateral ones, sternal shield deeply excavated posteriorly	***J.excavatus* Fain, 1987**
–	Dorsal shield with 20 pairs of setae (including eight opisthonotal pairs) heterogeneous in length, sternal shield straight posteriorly	***J.serratus* Maes, 1983**
10	Dorsal shield with 15 pairs of setae (including four opisthonotal pairs)	***J.celestiae* Uppstrom & Klompen, 2005**
–	Dorsal shield with more than 15 pairs of setae	**11**
11	Dorsal shield with 20 pairs of minute setae (including eight opisthonotal pairs)	***J.myriapodalis* Ryke, 1959**
–	Dorsal shield with 22–25 pairs of setae	**12**
12	Dorsal shield with 25 pairs of short setae, peritreme almost reaching to mid-level of coxae I	***J.cameroonensis* Maes, 1983**
–	Dorsal shield with 22, peritreme almost reaching anterior margin of coxae II	***J.vandaelensis* Maes, 1983**
13	Dorsal shield with 20 pairs of dorsal setae, setae *j4* and *z4* slightly longer than other dorsomedial setae	***J.parvitergalis* Ishikawa, 1986**
–	Dorsal shield with more than 20 pairs of setae, setae *j4*, *z4* conspicuously longer than other dorsomedial setae	**14**
14	Dorsal shield with 22 pairs of setae, female cheliceral fixed digit with two large teeth in addition to six-minute teeth	***J.nishikawai* Ishikawa, 1986**
–	Dorsal shield with 23 pairs of setae, female cheliceral fixed digit with three large teeth in addition to three small teeth	***J.parvungulatus* Ishikawa, 1986**
15	Epigynal shield drop-shaped, with an obvious (narrow) neck at level of coxae IV, narrower than anal shield	***J.luctator* Berlese, 1916**
–	Epigynal shield well developed, wider than anal shield	**16**
16	Dorsal shield with 39–40 pairs of setae; setae *j1* and *Z5* conspicuously longer than other dorsal setae; setae *z1* and *z6* present	**17**
–	Dorsal shield with 30–36 pairs of setae; setae *z1* and/or *z6* present or absent	**19**
17	Anal shield approximately twice as long as wide; para-anal setae at level of anterior margin of anus	***J.dispar* Berlese, 1916**
–	Anal shield nearly as long as wide; para-anal setae at level of posterior margin of anus	**18**
18	Setae *j1* and *Z5* subequal in length and longest dorsal setae; presternal platelets absent; femur IV with one thickened dorsal seta, idiosoma 1180 long, 770 wide	***J.pararotundatus* Ryke, 1959**
–	Setae *j1* shorter than *Z5*; *Z5* longest dorsal setae; presternal platelets present; femur IV with two thickened dorsal setae; idiosoma 1000–1012 long, 600 wide	***J.spirostrepti* Oudemans, 1914**
19	Dorsal shield with 36 pairs of setae; setae *z1*, *z6* and *S1* always present; setae *Z5* twice as long as *j1*	***J.moseri* Hunter & Rosario, 1986**
–	Dorsal shield with 32–33 pairs of setae; setae *z1*, *z6*, *r4*, *r6* absent and *S1* present or absent	**20**
20	Dorsal shield with 32 pairs of setae; *S1* absent; tritosternal base with ventral disc-like structure	***J.tritosternalis* Moraza & Kazemi, 2012**
–	Dorsal shield with 33 pairs of setae; *S1* present; tritosternal base normal and lacks ventral disc-like structure	***J.hallidayi* Nemati, Riahi & Gwiazdowicz, 2015**

### ﻿Modified key couplet to the species of *Julolaelaps* based on known males (after Moraza and Kazemi 2012)

**Table:** 

14	Dorsal shield with 22 pairs of setae; peritreme reaching the middle of coxae II	**15**
–	Dorsal shield with 25 pairs of short setae, peritremes extending to the middle of coxae I	***J.cameroonensis* Maes, 1983**
15	Sternal, genital, endopodal, ventral and anal shields fused into narrow holoventral shield	***J.vandaelensis* Maes, 1983**
–	Sternogenital and anal shields obviously separated	***Julolaelapsgigas* sp. nov.**

Prior to this study, three species of *Julolaelaps* were reported on the African giant millipede, *Archispirostreptusgigas* (Peters) (Farfan and Klompen 2012): *J.celestiae*, *J.kilifiensis* and *J.moseri*. The native range of *A.gigas* is in Africa, and the species has been moved throughout the world as part of the pet trade. So, if mites in this genus are host-specific, *Julolaelapsgigas* must also be of African origin, and has been moved around the world on its host. But most species of the genus have been collected on only a few occasions, and the host species have not been determined at the species level, making it difficult to draw firm conclusions about their host specificity. The question of host specificity of the species cannot be analyzed in detail until all available collections are re-examined to confirm their identification. On the contrary, Farfan and Klompen (2012) highlighted a hypothesis suggesting that the presence of these mites on millipedes might be linked to geographical location rather than host specificity. This implies that these mites could be specific to certain areas or types of off-host habitats. In this scenario, a diverse range of hosts within the preferred habitat could serve as suitable phoretic hosts. The intercontinental exchange and trade of millipedes, accompanied by the transportation of mites and potential millipede contamination, complicates our ability to ascertain the true origin of this mite species.

The millipede from which the mites were collected was obtained from a pet market where the import and breeding of exotic animals flourish as a business. The second author observed that the millipede was mite-free when it got to the Silkworm and Insect Management Center (Sangju, Republic of Korea) for an exhibition. Nonetheless, all specimens of *Julolaelapsgigas* were discovered on captive giant African millipedes. The mystery remains regarding how these mites came to inhabit this particular species. We need to do experiments to understand the real ecological role of these mites, regardless of where they came from.

## Supplementary Material

XML Treatment for Julolaelaps

XML Treatment for Julolaelaps gigas

## ﻿Additional information

### Author ORCIDs

Omid Joharchi http://orcid.org/0000-0002-2741-4946

Seoyoung Keum http://orcid.org/0000-0002-2219-9423

Chuleui Jung http://orcid.org/0000-0001-8134-9279

### Data availability

All of the data that support the findings of this study are available in the main text.

## References

[B1] Athias-Henriot C (1971) La divergence néotaxique des Gamasides (Arachnides). Bulletin Scientifique de Bourgogne 28: 93–106.

[B2] Athias-Henriot C (1975) Nouvelles notes sur les Amblyseiini. 2. Le relevé organotaxique de la face dorsale adulte (gamasides, protoadéniques, Phytoseiidae). Acarologia 17: 20–29.

[B3] Berlese A (1916) Centuria prima di Acari nuovi. Redia (Firenze) 12: 31–32.

[B4] Canestrini G (1891) Abbozzo del Sistema acarologico. Atti del Reale Istituto Veneto de Scienze, Lettere ed Arti 38(2): 699–725. 10.1007/BF03017253

[B5] Casanueva ME (1993) Phylogenetic studies of the free-living and arthropod associated Laelapidae (Acari: Mesostigmata). Gayana. Zoología 57: 21–46.

[B6] Downs WG (1943) Polyvinyl alcohol: A medium for mounting and clearing biological specimens. Science 97(2528): 539–540. 10.1126/science.97.2528.539 17832718

[B7] Evans GO (1963a) Observations on the chaetotaxy of the legs in the free-living Gamasina (Acari: Mesostigmata). Bulletin of the British Museum (Natural History). Zoology (Jena, Germany) 10(5): 275–303. 10.5962/bhl.part.20528

[B8] Evans GO (1963b) Some observations on the chaetotaxy of the pedipalps in the Mesostigmata (Acari). Annals and Magazine of Natural History (Series 13) 6: 513–527. 10.1080/00222936308651393

[B9] Evans GO Till WM (1979) Mesostigmatic mites of Britain and Ireland (Chelicerata: Acari-Parasitiformes). An introduction to their external morphology and classification. Transactions of the Zoological Society of London 35: 139–270. 10.1111/j.1096-3642.1979.tb00059.x

[B10] Fain A (1987) Notes on mites associated with Myriapoda. II. Four new species of the genus *Julolaelaps* Berlese, 1916 (Acari, Laelapidae). Bulletin de l’Institut Royal des Sciences Naturelles de Belgique Entomologie 57: 203–208.

[B11] Farfan M Klompen H (2012) Phoretic mite associates of millipedes (Diplopoda: Julidae) in the northern Atlantic region (North America, Europe). International Journal of Myriapodology 7: 69–91. 10.3897/ijm.7.3064

[B12] Gerdeman BS Klompen H Tanigoshi L (2000) Insights into the biology of a mite-millipede association. Fragmenta Faunistica 43: 223–227.

[B13] Hunter PE Rosario RMT (1986) A new species of the genus *Julolaelaps* Berlese (Acarina, Laelapidae). International Journal of Acarology 12(2): 63–67. 10.1080/01647958608683442

[B14] Ishikawa K (1986) Gamasid mites (Acarina) associated with Japanese millipeds. Reports of Research Matsuyama Shinonome Jr. College 17: 165–177.

[B15] Joharchi O Jung C Keum E (2018) First record of the genus *Myrmozercon* Berlese (Acari: Mesostigmata: Laelapidae) in the Eastern Palearctic region and description of a new species. International Journal of Acarology 44(7): 310–314. 10.1080/01647954.2018.1520298

[B16] Joharchi O Jung C Keum E (2019) New records of *Gaeolaelaps* Evans and Till (Mesostigmata: Laelapidae) from Republic of Korea with redescription of two species. International Journal of Acarology 45(8): 477–487. 10.1080/01647954.2019.1684990

[B17] Johnston DE Moraza ML (1991) The idiosomal adenotaxy and poroidotaxy of Zerconidae (Mesostigmata: Zerconina). In: Dusbábek F Bukva V (Eds) Modern Acarology.Academia, Prague, 349–356.

[B18] Keum E Kazmarek S Jung C (2016) A new record of *Hypoaspissardous* (Canestrini, 1884) (Acari: Mesostigmata: Laelapidae) from Korea. Journal of Species Research 5(3): 477–482. 10.12651/JSR.2016.5.3.477

[B19] Keum E Jung C Joharchi O (2017) New species and new records of the family Laelapidae (Acari: Mesostigmata) from Republic of Korea. Zootaxa 4353(3): 485–505. 10.11646/zootaxa.4353.3.5 29245499

[B20] Kontschán J (2005) Two species of *Julolaelaps* Berlese, 1916 (Acari: Mesostigmata: Laelapidae) associated with millipedes from Kenya. Annales Historico-Naturales Musei Nationalis Hungarici 97: 257–260.

[B21] Kontschán J Jeon MJ Hwang JM Seo HY (2015) New records to the Korean soil dwelling Mesostigmata fauna (Acari). Journal of Species Research 4(1): 33–44. 10.12651/JSR.2015.4.1.033

[B22] Kontschán J Hwang JM Jeon MJ Seo HY (2016) New Data to the Mite Fauna of the Korean Peninsula. StormingBrain, Budapest, 93 pp.

[B23] Kramer P (1886) Ueber Milben. I Zur Kenntnis einiger Gamasiden. Archiv für Naturgeschichte 52: 241–268. 10.5962/bhl.part.28438

[B24] Lindquist EE Evans GO (1965) Taxonomic concepts in the Ascidae, with a modified setal nomenclature for the idiosoma of the Gamasina (Acarina: Mesostigmata). Memoirs of the Entomological Society of Canada 47(S47): 1–64. 10.4039/entm9747fv

[B25] Maes K (1983) Scientific reports of the Belgian Mount Cameroon expedition 1981. VIII. Description of four new species of the genus *Julolaelaps* (Acarina: Laelapidae). Revue de Zoologie Africaine 97(1): 211–220.

[B26] Moraes GJ Moreira GF Freire RAP Beaulieu F Klompen H Halliday B (2022) Catalogue of the free-living and arthropod-associated Laelapidae Canestrini (Acari: Mesostigmata), with revised generic concepts and a key to genera. Zootaxa 5184(1): 1–509. 10.11646/zootaxa.5184.1.1 37044815

[B27] Moraza ML Kazemi S (2012) Description of a new millipede-associated species (Acari: Mesostigmata: Laelapidae) from Iran and a key to species of *Julolaelaps* Berlese. International Journal of Acarology 38(1): 6–17. 10.1080/01647954.2011.583273

[B28] Nemati A Riahi E Gwiazdowicz DJ (2015) Description of a new species of *Julolaelaps* (Acari, Mesostigmata, Laelapidae) from Iran. ZooKeys 526: 105–116. 10.3897/zookeys.526.5946 PMC4607847 26487827

[B29] Oh J Lee S Joharchi O (2023) A new species of *Gaeolaelaps* Evans & Till (Acari: Laelapidae) associated with an endemic bess beetle (Coleoptera: Passalidae) in the Republic of Korea. Acarologia 63(3): 676–690. 10.24349/m7r8-d4fp

[B30] Oudemans AC (1914) Acarologische Aanteekeningen. 52. Entomologische Berichten 4: 65–73.

[B31] Ryke PAJ (1959) A revision of the hypoaspid mites associated with Myriapoda with descriptions of three new species of the subgenus Julolaelaps Berlese (Acarina: Laelaptidae). Parasitology 49(1–2): 6–22. 10.1017/S0031182000026676 13657513

[B32] Salmane I Telnov D (2007) Laelaptidae mites (Parasitiformes, Mesostigmata) of east African millipedes (Diplopoda). Latvijas Entomologs 44: 121.

[B33] Swafford L Bond JE (2010) Failure to cospeciate: An unsorted tale of millipedes and mites. Biological Journal of the Linnean Society. Linnean Society of London 101(2): 272–287. 10.1111/j.1095-8312.2010.01499.x

[B34] Uppstrom K Klompen H (2005) A new species of *Julolaelaps* (Acari: Iphiopsididae) from African millipedes. International Journal of Acarology 31(2): 143–147. 10.1080/01647950508683666

[B35] Vitzthum H (1940–1943) Acarina – 5 Lieferung. In: H.G. Bronns. Klassen und Ordnungen des Tierreichs 5 (Abteilung IV, n.5): 1–1011.

